# Efficacy of topical and systemic treatments for atopic dermatitis on pruritus: A systematic literature review and meta-analysis

**DOI:** 10.3389/fmed.2022.1079323

**Published:** 2022-12-22

**Authors:** Youna Rodriguez-Le Roy, Anne-Sophie Ficheux, Laurent Misery, Emilie Brenaut

**Affiliations:** ^1^Department of Dermatology, University Hospital of Brest, Brest, France; ^2^Université de Bretagne Occidentale, LIEN, Brest, France

**Keywords:** pruritus, atopic dermatitis, systematic review, meta-analysis, itch, treatment

## Abstract

**Introduction:**

Pruritus is a major and burdensome symptom in atopic dermatitis (AD). The number of systemic treatments available for AD has increased recently, enabling improved patient relief.

**Objective:**

To evaluate the effect of AD treatments on pruritus.

**Methods:**

A systematic literature review and a meta-analysis were conducted to evaluate and compare the effects of treatment used in AD on pruritus. PubMed and Embase databases were searched to find articles published between January 1990 and December 2021. Topical and systemic treatments were studied in patients aged ≥10 years.

**Results:**

Among the 448 articles identified, 56 studies were retained in the systematic review. A total of 15 studies evaluated topical treatments: topical corticosteroids (TCS; 2), calcineurin inhibitors (6), PDE4 inhibitors (3), and Jak inhibitors (4). A total of five studies were included in the meta- analysis. All treatments had a positive effect on pruritus, with a mean overall reduction of 3.32/10, 95% IC [2.32–4.33]. The greatest reduction was observed with halometasone (mean: 4.75), followed by tofacitinib 2% (mean: 4.38). A total of 41 studies evaluated systemic therapies: cyclosporine (6), phototherapy (5), azathioprine (2), dupilumab (9), anti-IL 13 (5), nemolizumab (3), Jak inhibitors (9), mepolizumab (1), and apremilast (1). A total of 17 studies were included in 2 meta-analyses according to the concomitant use or not of TCS. In the meta-analysis without TCS, the overall decrease was 3.07/10, 95% IC [2.58–3.56]. The molecules with the highest efficacy on pruritus were upadacitinib 30 mg (mean: 4.90) and nemolizumab (mean: 4.81).

**Discussion:**

The therapeutic arsenal for AD has increased rapidly, and many molecules are under development. The primary endpoint of clinical trials is most often a score that assesses the severity of AD; however, the assessment of pruritus is also essential. The majority of molecules have a positive effect on pruritus, but the improvement varies between them. Efficacy on pruritus is not always correlated with efficacy on AD lesions; therefore, these two criteria are crucial to evaluate. The limitations of this study were the heterogeneity in the assessment of pruritus, the moment of the assessment, and the concomitant application of TCS or not for studies evaluating systemics. In the future, it would be useful to use standardized criteria for assessing pruritus.

## Introduction

Atopic dermatitis (AD) is a chronic, complex, relapsing, inflammatory skin disease characterized by xerosis, eczematous lesions, and pruritus. Its pathogenesis probably results from an interaction between skin barrier defects and immune dysregulation (1, 2). Although AD is recognized as a disease of children, increasing evidence suggests that it is more common in adults than previously thought (3). AD affects up to 20% of children worldwide (4) and from 2.1 to 4.9% of adults across industrialized countries (3).

The hallmark symptom of AD is pruritus (5), which is also its most common and burdensome symptom (6). The intensity of pruritus seems to be correlated with the severity of AD and tends to appear more frequently in the evening and at night (7). Pruritus causes difficulty in falling asleep in many patients with AD and is a leading cause of reduced health-related quality of life (8).

For a long time, few therapies were available for treating AD; topical treatments comprised topical steroids and tacrolimus/pimecrolimus, and few systemic treatments (9). Recently, however, new molecules have been commercialized and other promising therapies are under development (10).

The primary endpoints in clinical trials are objective scores of AD, such as the Eczema Area and Severity Index (EASI) (11). In the EASI score, there is no question about itch, which can be assessed indirectly by the items “excoriation” and “lichenification.” In the SCORing Atopic Dermatitis (SCORAD) Index, mean pruritus in the last 3 days is evaluated (12). Pruritus, evaluated using a visual analog scale (VAS) or a numerical rating scale (NRS), generally appears as a secondary criterion (11). In 2019, experts on AD gathered and published a paper with proposals of measurement instruments in AD (10). Furthermore, the efficacy of treatments on eczema lesions is not necessarily parallel to their effect on pruritus. From a patient’s perspective, pruritus is a major preoccupation (13), which is why AD treatments should also be directed toward the resolution of pruritus (14).

The aim of this study was to conduct a systematic review of the literature as well as a meta-analysis to evaluate and compare the effects of treatments used in AD on pruritus.

## Methods

A systematic literature search was performed in January 2022. The PubMed and Embase databases were searched to find clinical trials that have evaluated treatments for AD, published from January 1990 to December 2021. The following search string was used: “dermatitis, atopic” AND “pruritus or itch” AND “treatments.” The search was limited to human clinical trials in the English, French, or Spanish language. Only clinical trials evaluating the efficacy of treatments on pruritus with an objective scale (VAS, NRS, or semiquantitative scale) were selected. The peak pruritus NRS (PP-NRS) is used to measure peak pruritus, or “worst” itch over the previous 24 h, on a scale from 0 to 10.

After duplicates were removed, relevant studies were initially selected by reviewing the title and abstract. With regard to the remaining studies, the full articles were read. All studies included patients aged ≥10 years. Topical and systemic treatments of AD were selected.

In the second stage, a meta-analysis was performed. The primary outcome was the improvement in pruritus score between the baseline and the moment of evaluation of the primary endpoint. Pruritus was assessed using different scores. Only randomized controlled trials that have evaluated pruritus with a VAS or an NRS of 0–100 or 0–10 were included, where 0 indicated “no pruritus” and 10 or 100 indicated “the worst pruritus imaginable.” When the data on pruritus were presented only in graphs, the mean and standard deviation values were estimated with the WebPlotDigitizer tool.

The meta-analysis was performed using the computer program Review Manager (RevMan; version 5.4, The Cochrane Collaboration, 2020). An inverse variance model was used. Heterogeneity was evaluated with Cochran’s *Q*-test and *I*^2^ value. In cases of an *I*^2^ value higher than 25%, a random effect model was used; *P*-values < 0.05 were considered significant.

## Results

**FIGURE 1:**
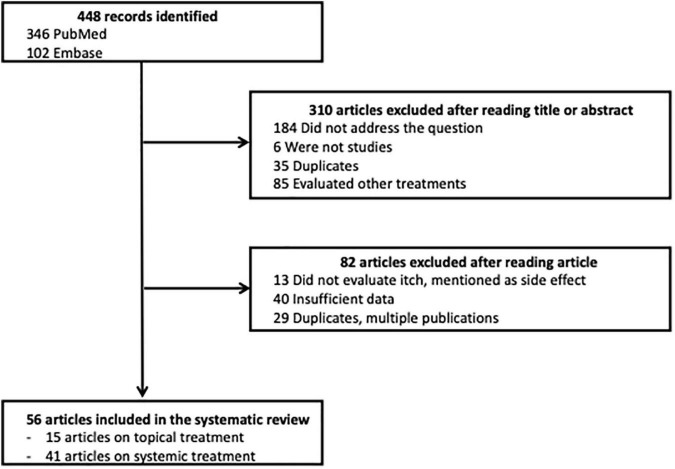
Flow chart.

Among the 448 articles identified, 310 articles were excluded after their title or abstract was read; 82 articles were excluded after the article was read; and 56 articles were selected for the systematic review, 15 about topical treatments and 41 about systemic treatments. A flow chart of this process is presented in Figure 1.

### Topical treatments (15 studies)

#### Topical corticosteroids (TCS; two studies)

In an open-label study, Yentzer et al. assessed a 3-day course of fluocinonide cream 0.1% applied twice daily on affected body surfaces (20 patients included) (15). At the end of the study (day 14), the VAS of pruritus had improved from baseline by 79% (from 5.6 to 1.2 on a scale from 0 to 10; *P* < 0.001).

In a prospective trial, wet-wrap therapy with halometasone cream was applied for 2 h twice daily for 7 days in 12 patients (16). Pruritus was assessed using an illustrated VAS with 10 color shades, where darkest shades represented the most severe itch. After the wet-wrap therapy, the mean VAS score of pruritus at baseline (7.5 ± 1.17) decreased significantly (2.75 ± 0.62; *P* < 0.001).

#### Tacrolimus (two studies)

In a double-blinded study, 3 arms were compared: Twice daily simultaneous application of desoximetasone 0.25% and tacrolimus 0.1% vs. tacrolimus 0.1% alone (82 patients included) (17). At the end of the treatment (day 21), the mean reduction in VAS score (0 to 3 from baseline) was significant: 2.11 in the single-active group and 2.37 in the double-active group (*P* = 0.006).

In another randomized study, Fleischer et al. compared tacrolimus ointment 0.1% with pimecrolimus cream 1% applied twice daily for up to 6 weeks or until 1 week after the affected area was completely cleared (281 patients included) (18). The baseline pruritus score was 6.7/10 in the 2 groups and the final scores were 3.2 and 3.8 in the tacrolimus and pimecrolimus groups, respectively (difference between arms not significant).

#### Pimecrolimus (four studies)

Meurer et al. studied the long-term effectiveness of pimecrolimus cream 1% in a 6-month placebo-controlled study (192 patients included) (19). Treatment was applied twice daily to treat the first signs or symptoms of AD as required over 24 weeks. Pruritus was evaluated during the first week of treatment. At day 3, the pruritus score decreased significantly more in the pimecrolimus group (from 2.5 at baseline to 2.1) than in placebo group (*P* < 0.001). This difference was still significant after 7 days of treatment; the pruritus score was 1.6 in the pimecrolimus group vs. 2.5 in the control group.

In a prospective, double-blinded, randomized study, the efficacy of pimecrolimus cream 1% applied twice daily (BID) or four times daily (QID) over 1 week was evaluated in 49 patients (20). After the first week, the patients used pimecrolimus cream twice daily with the option of applying 2 further daily treatments (active or vehicle) for 2 weeks. Pruritus was assessed using a four-point scale (0–3). At baseline, the pruritus scores were similar between the two groups. Both the BID and QID treatment regimens were rapid and effective in controlling pruritus; no significant difference existed between the two groups in terms of the percentage of patients who reported their pruritus to be either absent or mild at the end of the study (52.9 and 50% in the QID and BID groups, respectively).

Kaufmann et al. studied pruritus relief with pimecrolimus cream 1% in a randomized, double-blinded, vehicle-controlled trial (198 patients included) (21). At baseline, 87% of the patients in the pimecrolimus group and 82% in the vehicle group suffered from moderate or severe pruritus (evaluated at 2 or 3). At day 7, 81% of the pimecrolimus-treated patients vs. 63% of vehicle-treated patients exhibited an improvement (≥1-point compared with baseline; *P* < 0.001).

Aschoff et al. evaluated skin physiological parameters to confirm the therapeutic efficacy of pimecrolimus cream 1% in patients with mild-to-moderate AD (22). Pimecrolimus was applied twice daily on one forearm and vehicle cream was applied on the other forearm (20 adults included). At the end of treatment (day 21), the pruritus score decreased from 4.5/10 to 1.0 in the pimecrolimus-treated forearm and from 4.5/10 to 2.5 in the vehicle-treated forearm (*P* = 0.006).

#### Phosphodiesterase type-4 inhibitor (PDE4) (three studies)

A randomized, vehicle-controlled trial evaluated the efficacy of topical E6005 2%, a PDE4 inhibitor, which was applied twice daily (23). A total of 78 patients were included and itch was evaluated using an itch behavioral rating scale (IBRS; 0–8 points). At week 12, the IBRS score was not significantly reduced in the treatment group compared with the placebo group (*P* = 0.462).

A novel, topical, non-steroidal, selective PDE4 inhibitor (OPA-15406) was evaluated in a phase II, randomized, double-blinded, placebo-controlled study (24). OPA-15406 at doses of 0.3% or 1% was applied twice daily across 8 weeks (121 patients included). The mean VAS pruritus scores within 1 week improved more in the OPA-15406 1% group (from 63.7 ± 20.3 to 40.5 ± 27.1) than in placebo group (*P* = 0.0011). At week 4, the decrease was 2.95 in the placebo group, 3.95 in the OPA 0.3% group, and 14.4 in the OPA 1% group (*P* = 0.0452).

In a phase II, single-center, randomized study, Bissonnette et al. assessed crisaborole ointment 2% or vehicle applied twice daily for 14 days (40 patients included) (25). An improvement in pruritus NRS score (11 points NRS) was observed in the crisaborole group compared with the vehicle group on the first day after treatment initiation (day 2: −1.9 vs. −1.0; *P* = 0.0188) with continued improvement through day 15 (−3.9 vs. −2.0; *P* < 0.0001).

#### Topical Janus kinase inhibitor (JAKi) (four studies)

Topical 2% tofacitinib was evaluated in a 4-week, phase II, double-blinded, vehicle-controlled, randomized trial (69 patients included) (26). Itch was measured using the itch severity item (ISI) scale, which ranges from 0 to 10. At baseline, the overall mean ISI score was 6.0.

At week 4, the ISI score had decreased by 4.45 in the tofacitinib group and by 1.15 in the vehicle group (*P* < 0.001). A significant improvement in pruritus was observed within 48 h of the first application of tofacitinib. Furthermore, the least-squares (LS) mean changes from baseline in ISI scores were significantly greater for tofacitinib vs. vehicle from day 2 through day 14 (*P* < 0.001) and at weeks 2 and 4 (*P* < 0.0001).

Nakagawa et al. assessed the efficacy and safety of topical JTE-052, a JAKi, in a phase II, randomized, vehicle-controlled study (27). JTE-052 ointment at 0.25, 0.5, 1, or 3% was compared with vehicle ointment or tacrolimus 0.1% ointment applied twice daily for 4 weeks (327 patients included). At the end of the treatment, the JTE-052 0.5, 1, and 3% groups exhibited significant improvements from baseline compared with the vehicle group in terms of the pruritus NRS score. Moreover, these scores over the first week exhibited improvements compared with placebo (*P* < 0.001) from day 1 in the JTE-052 0.5% (−1.29), 1% (−0.99), and 3% groups (−1.97) as well as in the tacrolimus group (−1.39).

Nakagawa et al. reported results for delgocitinib 0.5% ointment, which they studied in a phase III, randomized, double-blinded, vehicle-controlled trial followed by an open-label, long-term extension study (158 patients included) (28). Pruritus was evaluated using a pruritus NRS (0–10). At the end of treatment, the pruritus score had decreased from 5.3 to 3.7 in the delgocitinib group and from 5.4 to 5.4 in the placebo group (*P* < 0.001). The daily changes in the score indicated a rapid reduction in pruritus after the treatment started in the delgocitinib group, which had a pruritus NRS score lower than that of the vehicle group at week 1; this difference was maintained over time. The authors’ long-term extension study revealed that long-term treatment with delgocitinib maintained the improvement in pruritus NRS scores.

The effect of ruxolitinib cream on pruritus was studied in a phase II, randomized, dose-ranging, vehicle-controlled study (307 patients included) (29). Ruxolitinib at different dosages (1.5% twice daily, 1.5% once daily, 0.5% once daily, or 0.15% once daily) was compared with vehicle twice daily over 8 weeks, or with triamcinolone cream (0.1% twice daily for 4 weeks followed by vehicle for 4 weeks). Decreases in itch NRS scores were noted within the first 2 weeks of treatment for all ruxolitinib cream groups and were sustained throughout the study. At week 4, both 1.5% ruxolitinib cream groups exhibited a more pronounced alleviation in itch compared with the triamcinolone group. The mean percentage change from baseline was −64.6 for the group administered 1.5% twice daily, −54.0 for the group administered 1.5% once daily, and −50.3 for the group administered triamcinolone. The difference was statistically significant for 1.5% ruxolitinib twice daily vs. triamcinolone (*P* = 0.003).

#### Meta-analysis on topical treatment

**FIGURE 2:**
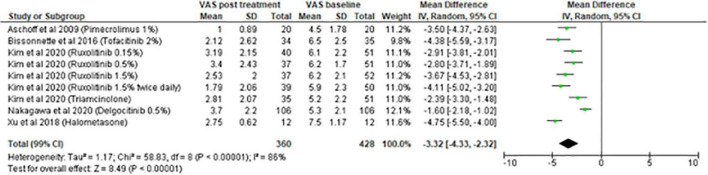
Meta-analysis on topical treatments (five studies).

The meta-analysis for topical treatments included five articles (Figure 2). The summary of the characteristics of the 5 studies included is presented in Table 1. All treatments—halometasone, triamcinolone, delgocitinib, ruxolitinib, and tofacitinib—had a beneficial impact on pruritus, with a mean reduction in pruritus score of 3.32 points (99% CI, [2.32–4.33]), the greatest effect was observed with halometasone in wet-wrap therapy.

**TABLE 1:** Summary of the characteristics of the studies included in the meta-analysis on topical treatments.

References	Treatment	Study design	Evaluation of pruritus	Results	Time of evaluation
Xu et al. (16)	Wet wrap therapy with halometasone applied twice daily, during 2 hours for 7 days	Single-center, prospective cohort study	VAS 0-10 cm	VAS score of pruritus at baseline : 7.5 ± 1.17 VAS score of pruritus at the end of treatment : 2.75 ± 0.62, *p* < 0.001	7 days
Aschoff et al. (22)	Pimecrolimus cream 1 % applied twice daily on one forearm and vehicle cream on the other forearm	Double-blind, randomized, within-patient placebo-controlled, single center study	VAS 0-10 cm	At baseline, Mean pruritus score for pimecrolimus : 4.5 Mean pruritus score for vehicle : 4.5 At day 21, Mean pruritus score for pimecrolimus : 1.0 Mean pruritus score for vehicle : 2.5, *p* = 0.006	21 days
Bissonnette et al. (26)	2% tofacitinib or vehicle ointment applied twice daily for 4 weeks.	Phase 2, multi-center, randomized (1:1), double-blind, vehicle-controlled, parallel-group study	Ich severity item score 0-10	At baseline, overall mean ISI score was 6.0 At week 4, the ISI score had decreased by 4.45 in the tofacitinib group and by 1.15 in the vehicle group (*p* < 0.001)	28 days
Nakagawa et al. (28)	Delgocitinib 0.5% ointment or vehicle ointment applied twice daily	Phase 3, randomized (2:1), double-blind, vehicle-controlled study	Pruritus NRS 0-10	At the end of treatment, the pruritus score had decreased from 5.3 to 3.7 in the delgocitinib group and from 5.4 to 5.4 in the placebo group (*P* < 0.001 compared with placebo)	28 days
Kim et al. (29)	Ruxolitinib (RUX) : 1.5% twice daily 1.5% once daily 0.5% once daily 0.15% once daily vehicle twice daily or triamcinolone cream 0.1% twice daily	Phase 2, randomized (1:1:1:1:1:1:), double blind, dose-ranging, vehicle-controlled, multicenter study	Itch NRS 0-10	At baseline, the mean itch NRS score was : Vehicle BID : 6.0 0.1% TAC BID : 5.2 RUX cream 0.15% QD : 6.1 RUX cream 0.5% QD : 6.2 RUX cream 1.5% QD : 6.2 RUX cream 1.5% BID : 5.9 At week 8, the mean itch NRS score was : 0.1% TAC BID : 2.81 RUX cream 0.15% QD : 3.19 RUX cream 0.5% QD : 3.4 RUX cream 1.5% QD : 2.53 RUX cream 1.5% BID : 1.79	56 days

### Systemic treatments

In total, 41 studies evaluated the efficacy of systemic treatments for AD on pruritus.

#### Cyclosporine (six studies)

In 1990, a randomized, placebo-controlled, crossover study evaluated the efficacy of cyclosporine (5 mg/kg/day) in 10 patients with moderate or severe AD suffering from distressing pruritus, which frequently disturbed their sleep (30). Cyclosporine significantly reduced the itch intensity (VAS 0–100 mm) on treatment days 9–10 compared with the baseline value (−19 mm, *P* = 0.02) as well as compared with days 9–10 of the placebo group (*P* < 0.01).

In a double-blinded, placebo-controlled, crossover study, Sowden et al. compared the effect of cyclosporine (5 mg/kg/day) vs. placebo in 33 patients (31). At week 8 (the end of the first period), the mean itch score in the placebo-cyclosporine sequence had decreased from 56.6 to 53.0 (VAS 0–100 mm) compared with 51.1 to 23.4 in the cyclosporine-placebo sequence (*P* < 0.001).

Munro et al. reported the results of a crossover, double-blinded, randomized, placebo-controlled study (24 patients were included). Cyclosporine (5 mg/kg/day) or placebo was administered across 8 weeks and the other treatment across the following 8 weeks (32). At baseline, the mean pruritus score (VAS 0–10 cm) was 5.8. At the end of treatment, mean itch score was 4.4 for placebo and 1.8 for cyclosporine (difference 2.8, 95% CI [1.8–3.8]).

Furthermore, 46 patients were included in a 6-week, double-blinded, placebo-controlled trial that evaluated cyclosporine (5 mg/kg/day) (33). At week 6, the mean pruritus score (0–3) in the cyclosporine-treated group decreased from 2.7 to 1.1, compared with 2.6 to 2.1 in the placebo group (*P* = 0.01).

In 1996, Zonneveld et al. assessed cyclosporine at a dose of 5 mg/kg per day decreased to 3 mg/kg per day (Group A) or a dose of 3 mg/kg per day increased to 5 mg/kg per day (Group B) (78 patients included) (34). After a 2-month dose-finding period, the patients were maintained on their optimal dose for 10 months. Itch was evaluated using a four-point scale (0–3). At baseline, 57% of the patients rated itch as severe while 40% rated it as moderate. After 2 weeks of cyclosporine therapy, only 5.5 and 28% of patients rated itch as severe and moderate, respectively, with an improvement significantly higher in group A (*P* = 0.015). Itch scores in both treatment groups continued to improve throughout the 10 months of treatment, but the differences between the groups were not statistically significant after week 2.

In a prospective open study that included 100 patients, Berth-Jones et al. evaluated the long-term efficacy of cyclosporine (35). The dose was 5 mg/kg/day for the first 8 weeks, which was then adjusted according to the patients’ response. Pruritus was evaluated using a four-point scale (0–3). At week 48, the mean pruritus score had decreased from 2.3 (baseline) to 1.6 (*P* < 0.0001).

#### Phototherapy (five studies)

In 1990, Jekler and Larko compared UVA + UVB radiation on one side of the body and UVB radiation on the other side in 30 patients (36). The patients underwent irradiation three times a week for a maximum of 8 weeks. Pruritus was evaluated using a four-point scale (0–3). At the end of treatment, the mean pruritus scores decreased from 2.4, to 1.2 and 1.0 for UVB and UVA + UVB therapy, respectively (*P* = 0.04).

In 1991, the same authors included 21 patients in a bilateral, randomized, left-right comparison trial. In one half of the body, patients were treated with UVA lamps, while in the other half they were treated with UVB lamps (37). Pruritus scores were evaluated using a four-point scale. After UVA therapy and UVB therapy, the mean pruritus scores decreased from 2.2 to 1.1 and 1.3, respectively, difference between the 2 types of UV.

In a randomized trial, Valkova et al. compared the efficacy of UVA + UVB therapy vs. UVA + UVB therapy combined with TCS (31 patients included). They demonstrated that pruritus diminished significantly in all patients in both groups (38).

In a randomized, double-blinded, controlled, crossover trial, the efficacy of medium-dose UVA1 vs. narrowband UVB phototherapy was evaluated (47 patients included) (39). At the end of the first period, pruritus had decreased from 6.12 ± 2.2 to 4.2 ± 2.42 in the UVA1 group and from 7.5 ± 2.1 to 4.5 ± 2.3 in the NB-UVB group. Although both interventions were associated with a significant improvement in pruritus, no significant difference existed between the treatments.

After narrowband UVB phototherapy, Väkevä et al. evaluated the therapy’s effect on pruritus over time in 144 patients (40). At baseline, the mean VAS pruritus score was 5.2, which diminished to 3.1 after therapy and to 2.5 3 months after therapy (*P* < 0.001). Furthermore, the initial pruritus VAS scores correlated highly significantly with the initial Dermatology Life Quality Index values (*P* < 0.001).

#### Azathioprine (two studies)

Berth-Jones et al. presented the results of a crossover, double-blinded, randomized, placebo-controlled trial of azathioprine administered at 2.5 mg/kg/day (37 patients included) (41). Pruritus was evaluated using a horizontal 10 cm VAS. At week 12 (end of the first period), the mean pruritus score in the azathioprine-placebo sequence had decreased from 4.5 to 3.0 compared with 4.5 to 3.9 in the placebo-azathioprine sequence.

Meggitt et al. evaluated the efficacy of azathioprine (42 patients) vs. placebo (21 patients) and a graduated-dose regimen based on azathioprine pharmacogenetics (42). Patients with heterozygous-range thiopurine methyltransferase (TPMT) activity received azathioprine at 1.0 mg/kg daily, compared with 2.5 mg/kg daily in patients with normal TPMT activity in the maintenance period (after 4 weeks). At week 12, the pruritus score (VAS 0–10) decreased from 5.4 to 3.0 in the azathioprine group and from 5.7 to 4.7 in the placebo group (difference 1.4, 95% CI [0.1–2.7]).

#### Dupilumab (nine studies)

Beck et al. presented the results of 4 studies with the same design (randomized, double-blinded, and placebo-controlled, with 207 patients included) (11). In 3 trials, dupilumab as a monotherapy was compared with placebo (two 4-week trials and one 12-week trial). In the fourth trial, dupilumab was used in combination with TCS (4-week trial). In the two 4-week trials, the mean pruritus score decreased from 6.0 to 3.5, whereas in the 12-week trial it decreased from 6.1 to 3.4, the efficacy of dupilumab on pruritus was superior to placebo (*P* < 0.001). In the fourth trial (dupilumab + TCS), the mean pruritus score decreased from 6.4 to 1.9.

In a dose-ranging study, Thaçi et al. compared dupilumab 100 mg every 4 weeks, 200 mg every 2 weeks, 300 mg every 2 weeks, 300 mg every 4 weeks, and 300 mg once weekly with placebo (379 patients included) (43). They demonstrated an improvement in weekly PP-NRS scores, which appeared to be dose-dependent. At week 16, all dupilumab dose regimens except for 100 mg/4 weeks resulted in LS mean percentage reductions in pruritus of approximately 33–47% (*P* < 0.0001 vs. placebo). The greatest improvement was reported for doses of 300 mg every 2 weeks and 300 mg once a week.

In a randomized, double-blinded, parallel-group, dose-ranging study, Simpson et al. compared dupilumab 100 mg every 4 weeks, 200 mg every 2 weeks, 300 mg every 4 weeks, 300 mg every 2 weeks, and 300 mg once weekly with placebo (380 patients included) (44). The mean pruritus score decreased significantly more for dupilumab than with placebo. The LS mean change from baseline was −0.1 for placebo, −1.1 for dupilumab 100 mg/4 weeks (*P* < 0.009), −2.4 for dupilumab 300 mg/4 weeks (*P* < 0.0001), −2.3 for dupilumab 200 mg/2 weeks (*P* < 0.0001), −2.8 for dupilumab 300 mg/2 weeks (*P* < 0.0001), and −3.2 for dupilumab 300 mg/week (*P* < 0.0001).

Simpson et al. reported results of 2 phase III trials with the same design of dupilumab vs. placebo (671 and 708 patients included, respectively) (45). The patients were randomly assigned in a 1:1:1 ratio to receive 300 mg dupilumab or placebo weekly for 16 weeks or the same dose of dupilumab every other week alternating with placebo. At week 16, an improvement of at least 3 or 4 points in the PP-NRS score occurred in significantly more patients receiving dupilumab than in those receiving placebo (*P* < 0.001).

The effect of dupilumab on pruritus over time was evaluated in a 1 year, randomized, double-blinded, placebo-controlled study that compared dupilumab 300 mg once weekly, dupilumab 300 mg every 2 weeks, or placebo with concomitant TCS (740 patients included) (46). At week 16, an itch response (defined as a PP-NRS improvement of 4 or higher) was achieved in 51% (150/295) of patients who received dupilumab weekly, in 59% (60/102) of those who received dupilumab every 2 weeks, and 20% (59/299) of those in the control group (*P* < 0.0001). At week 52, an itch response was achieved in 39% of patients treated with dupilumab weekly, in 51% treated with dupilumab every 2 weeks, and in 13% treated with placebo (*P* < 0.0001).

In a randomized, double-blinded, placebo-controlled study, de Bruin Weller et al. studied dupilumab 300 mg weekly or every 2 weeks with concomitant TCS (325 patients included) across 16 weeks (47). Pruritus was evaluated using a weekly average of daily PP-NRS scores. Dupilumab + TCS significantly improved scores from baseline to week 16 compared with placebo + TCS. At week 16, the LS mean percentage change from baseline was −25.4, −53.9, and −51.7 for placebo, dupilumab every 2 weeks, and dupilumab weekly, respectively (*P* < 0.001 for dupilumab groups vs. placebo).

In a randomized, double-blinded, placebo-controlled trial, Tsianakas et al. tested dupilumab at a dose of 300 mg weekly for 12 weeks in 64 patients (48). At baseline, the mean pruritus NRS score (0–10) was 5.7 in the dupilumab arm and 5.5 in the placebo arm. At week 12, dupilumab had significantly reduced pruritus score compared with placebo (*P* < 0.001). The LS mean percentage difference vs. placebo was 50.5 ± 9.27%.

Simpson et al. studied the efficacy of dupilumab in adolescents with uncontrolled moderate-to-severe AD (49). Patients were randomized (1:1:1) to a 16-week treatment with dupilumab 200 mg (if baseline weight <60 kg) or dupilumab 300 mg (if baseline weight ≥60 kg) every 2 weeks or dupilumab 300 mg every 4 weeks or placebo (251 patients included). A significant improvement was observed in the LS mean percentage change from baseline to week 16 in the PP-NRS (0–10): −47.9 for dupilumab every 2 weeks, −45.5 for dupilumab every 4 weeks, and −19.0 for placebo.

In a randomized, double-blinded, placebo-controlled trial, Zhao et al. included 165 patients receiving 300 mg of dupilumab or placebo every 2 weeks for 16 weeks (50). At week 16, significantly higher proportions of patients in the dupilumab group than in the placebo group had ≥3-points (52.4 vs. 9.6%) and ≥4-points (39.0 vs. 4.8%) reductions in weekly average PP-NRS scores (*P* < 0.001 for both endpoints). Furthermore, the proportions of patients who had ≥3- and ≥4-points reductions in said scores were greater with dupilumab than with placebo at all assessment timespoints from week 2.

#### Anti-IL-13 monoclonal antibodies (five studies: three with tralokinumab, two with lebrikizumab)

Tralokinumab (doses of 45, 150, or 300 mg every 2 weeks) was compared with a placebo in a randomized, double-blinded, dose-ranging study of 12 weeks, with the concomitant use of TCS (204 patients included) (51). At week 12, the mean pruritus score (measured with a pruritus NRS ranging from 0 to 10) decreased for placebo (−1.00), tralokinumab 45 mg (−1.77), tralokinumab 150 mg (−1.59), and tralokinumab 300 mg (−2.14). The difference was significant for tralokinumab 45 and 300 mg compared with placebo.

Wollenberg et al. presented results from two 52-week randomized, double-blinded, placebo-controlled, phase III trials named ECZTRA 1 and ECZTRA 2 (802 patients). Adults were randomized (3:1) to subcutaneous tralokinumab 300 mg every 2 weeks (52). A reduction in weekly average worst daily pruritus NRS scores (0–10) by ≥4-points from baseline to week 16 was achieved by 20.0% of patients receiving tralokinumab vs. 10.3% receiving placebo in ECZTRA 1 (difference 9.7, 95% CI [4.4–15.0]; *P* = 0.002) and by 25.0 vs. 9.5% in ECZTRA 2 (difference 15.6, 95% CI [10.3–20.9]; *P* < 0.001).

Silverberg et al. studied tralokinumab plus TCS in a double-blinded, randomized, multicenter, placebo-controlled phase III trial (380 patients included) (53). Adults were randomized 2:1 to subcutaneous tralokinumab 300 mg every 2 weeks with TCS as required over 16 weeks. At week 16, a significantly greater proportion of patients treated with tralokinumab vs. those treated with placebo achieved a ≥4-points reduction in weekly average worst daily pruritus NRS scores (0–10) (45.4 vs. 34.1% [11.3% (0.9–21.6); *P* = 0.037]).

Simpson et al. compared lebrikizumab 125 mg single dose, lebrikizumab 250 mg single dose, lebrikizumab 125 mg every 4 weeks for 12 weeks, or placebo every 4 weeks for 12 weeks after a 2-week TCS run-in (209 patients were randomized at a 1:1:1:1 ratio) (54). The mean percentage reduction in baseline pruritus VAS score (0–10) was 34.9% in the lebrikizumab 125 mg single-dose group, 32.8% in the lebrikizumab 250 mg single-dose group, and 40.7% in the lebrikizumab 125 mg/4 weeks group. The placebo group also exhibited reductions from baseline pruritus VAS score (27.5%), which resulted in the placebo-corrected efficacy not being statistically significant.

Guttman-Yassky et al. assessed the efficacy of lebrikizumab at doses of 125 mg every 4 weeks, 250 mg every 4 weeks, or 250 mg every 2 weeks vs. placebo (280 patients included) (55). Pruritus was evaluated using an NRS ranging from 0 to 10 (worst itch in the prior 24 h). The lebrikizumab groups exhibited dose-dependent, statistically significant improvements in LS mean percentage change from baseline in pruritus NRS score at week 16 vs. the placebo group: −4.3% for placebo, −35.9% for lebrikizumab 125 mg/4 weeks (*P* = 0,005), −49.6% for lebrikizumab 250 mg/4 weeks (*P* < 0.001), and −60.6% for lebrikizumab 250 mg/2 weeks (*P* < 0.001).

#### Nemolizumab (three studies)

Ruzicka et al. evaluated nemolizumab at different doses of 0.1, 0.5, 2.0 mg/kg, or placebo every 4 weeks or an exploratory dose of 2.0 mg/kg every 8 weeks (264 patients included) (56). A VAS (0–100 mm) was used to assess pruritus (primary endpoint). At week 12, among patients who received nemolizumab every 4 weeks, a significant, dose-dependent reduction in the LS mean percentage change from baseline in pruritus VAS scores was noted compared with placebo. The percentage reductions were −43.7% in the 0.1 mg/kg group, −59.8% in the 0.5 mg/kg group, and −63.1% in the 2.0 mg/kg group, vs. −20.9% in the placebo group (*P* < 0.01 for all comparisons).

Nemolizumab was studied in another phase II trial at doses of 10, 30, and 90 mg every 4 weeks vs. placebo with TCS (226 patients included) (57). All doses of nemolizumab were associated with a rapid decrease in pruritus scores (scale 0–10), with statistically significant differences from placebo starting as early as week 1. At week 2, scores with all nemolizumab doses were lower than those with placebo (*P* ≤ 0.001), and these results were maintained throughout the study. The most marked effects were observed in the 30 mg nemolizumab arm compared with the placebo arm (−67.3 vs. −35.8% at week 24; *P* < 0.001).

In a phase III trial, Kabashima et al. assessed the efficacy on the pruritus score (primary endpoint) of nemolizumab 60 mg every 4 weeks over 16 weeks (215 patients included) (58). The use of concomitant topical agents was allowed. The median VAS score (0–100) for pruritus at baseline was 75. At week 16, the mean percent change in the VAS score was −42.8 ± 2.6% in the nemolizumab group and −21.4 ± 3.6% in the placebo group (*P* < 0.001).

#### Systemic Janus kinase inhibitor (JAKi) (nine studies: one with baricitinib, four with abrocitinib, three with upadacitinib, and one with ASN002)

Guttman-Yassky et al. compared once-daily baricitinib 2, 4 mg, or placebo in a 16-week, randomized, double-blinded study (124 patients included) (59). Before randomization, the patients applied a TCS for 4 weeks, which could be used throughout the study. Itch was evaluated using an NRS ranging from 0 to 10. At week 4, the change from baseline was significant for both baricitinib groups compared with placebo (−0.8 for placebo, −2.7 for baricitinib 2 mg, and −3.1 for baricitinib 4 mg; *P* < 0.001). At the end of treatment (week 16), the change from baseline was non-significant compared with placebo (−1.7 for placebo, −2.6 for baricitinib 2 mg, and −2.2 for baricitinib 4 mg).

In a phase II, placebo-controlled, randomized trial, Gooderham et al. compared abrocitinib at different dosages of 200, 100, 30, or 10 mg administered once daily (267 patients included) (60). At week 12, significant reductions in pruritus NRS scores (0–10) were observed in the 200 mg (−25.4%, *P* = 0.003) and 100 mg (−20.7%, *P* = 0.02) groups compared with placebo.

In a randomized, double-blinded, phase III trial, Simpson et al. evaluated the efficacy of abrocitinib 100 mg, abrocitinib 200 mg, or placebo once daily for 12 weeks (387 patients included) (61). Pruritus was evaluated using a PP-NRS ranging from 0 to 10 and a response was defined as an improvement ≥4-points from baseline. The proportion of patients achieving a PP-NRS response increased between weeks 2 and 12 for both abrocitinib groups, with significant differences identified between the abrocitinib groups and placebo at weeks 2, 4, and 12. PP-NRS scores decreased between baseline and week 12: 15% of responders in the placebo group, 38% in the abrocitinib 100 mg group, and 57% in the abrocitinib 200 mg group (differences significant compared with placebo, respectively, *p* = 0.0003 and *p* < 0.0001).

In a phase III, double-blinded, randomized trial, Silverberg et al. evaluated daily abrocitinib 100 mg, abrocitinib 200 mg, or placebo for 12 weeks (391 patients included) (62). At week 12, 55.3% of patients achieved a PP-NRS response in the 200 mg abrocitinib group, 45.2% in the 100 mg abrocitinib group, and 11.5% in the placebo group (*P* < 0.001). Decreases from baseline in PP-NRS scores were greater in the abrocitinib groups than in the placebo group at all timespoints.

In a 16-week, randomized, double-blinded study, Bieber et al. compared abrocitinib 200 mg once daily, abrocitinib 100 mg once daily, and dupilumab 300 mg every week with placebo (838 patients included) (63). Pruritus was evaluated using a PP-NRS ranging from 0 to 10. An itch response (defined as a ≥4-points improvement from baseline) at week 2 occurred in 49.1% of patients in the 200 mg abrocitinib group, in 31.8% of patients in the 100 mg abrocitinib group, in 26.4% of patients in the dupilumab group, and in 13.8% patients in the placebo group. The 200 mg dose of abrocitinib, but not the 100 mg dose, was significantly superior to dupilumab with respect to itch response at week 2. At week 16, neither abrocitinib doses differed significantly from dupilumab.

Guttman-Yassky et al. assessed once-daily upadacitinib at 7.5, 15, or 30 mg vs. placebo (167 patients included) (64). Each upadacitinib dose level was significantly superior to placebo in terms of patient improvement in pruritus NRS (ranging from 0 to 10). Indeed, the mean improvement from baseline to week 16 was 73% in the 30 mg upadacitinib group (*P* < 0.001), 53% in the 15 mg upadacitinib group (*P* < 0.001), and 46% in the 7.5 mg upadacitinib group (*P* < 0.01) vs. 20% in the placebo group.

In a randomized, placebo-controlled trial, Reich et al. evaluated the efficacy of upadacitinib 15 or 30 mg in combination with TCS (901 patients included) (65). Pruritus was evaluated using a weekly average of a worst pruritus NRS (WP-NRS) ranging from 0 to 10. The proportion of patients who achieved at least a four-point improvement in WP-NRS score at week 1 was significantly higher in the upadacitinib groups than in the placebo group (*P* < 0.0001 for both doses). The proportion of patients who achieved an itch reduction at week 4 continued to increase between week 4 and 12 and was maintained until week 16. At week 16, 51.7% of patients were WP-NRS responders in the upadacitinib 15 mg + TCS arm, 63.9% in the upadacitinib 30 mg + TCS arm, and 15% in the placebo once daily + TCS arm (*P* < 0.0001 for both doses of upadacitinib compared with placebo).

In a 24-week, randomized, double-blinded trial, Blauvelt et al. compared the efficacy of upadacitinib 30 mg once daily vs. dupilumab 300 mg every 2 weeks (692 patients included) (66). The mean percentage improvement from baseline WP-NRS (scale 0–10) was significantly greater in the upadacitinib group compared with the dupilumab group as early as week 1 (31.4 vs. 8.8%; *P* < 0.001) and week 4 (59.5 vs. 31.7%; *P* < 0.001), and significant differences were maintained through week 16 (66.9 vs. 49.0%; *P* < 0.001). Furthermore, the proportion of patients achieving a clinically meaningful improvement in itch (defined as a WP-NRS improvement ≥4-points from baseline) at week 16 was higher for those receiving upadacitinib than for those receiving dupilumab: 55.3 vs. 35.7%, respectively; *P* < 0.001).

In a phase Ib, randomized, double-blinded, placebo-controlled study, Bissonnette et al. reported the results of an oral Janus kinase/spleen tyrosine kinase inhibitor (ASN002) compared with placebo (67). Three doses were studied over a 28−day period (20, 40, and 80 mg once daily) and 36 patients were included. At day 29, among patients with a baseline weekly average pruritus NRS score of at least 4, pruritus decreased significantly more in patients receiving ASN002 80 mg (−4.7 ± 2.1) than in those receiving placebo (−1.6 ± 1.8), *P* = 0.01. The difference was not statistically significant for patients receiving ASN002 40 or 20 mg.

#### Mepolizumab (one study)

Mepolizumab (2 single doses of 750 mg) was evaluated in a randomized, placebo-controlled trial that included 40 patients (68). At baseline, the mean VAS pruritus score was 5.6/10 in the mepolizumab group and 5.5/10 in the placebo group. At day 14, mean VAS pruritus score decreased more in the mepolizumab group (−2.6 cm) than in the placebo group (−1.3 cm), but the difference was non-significant. In addition, no clinical success was reached in the Physician Global Assessment score (*P* = 0.115) or SCORAD (*P* = 0.293) in the mepolizumab group compared with placebo.

#### Apremilast (one study)

In an open-label pilot study, Samrao et al. evaluated apremilast 20 mg twice daily over 3 months (cohort 1: 6 patients) and apremilast 30 mg twice daily over 6 months (cohort 2: 10 patients) (69). The patients in cohort 1 experienced an average reduction in itch of 49% using a VAS (0–100 mm) from a mean baseline of 62.3 to 30.5 mm, and a 25% reduction in cohort 2 from 45.8 to 32.4 mm (*P* = 0.02 and *P* = 0.03, respectively).

#### Meta-analysis on systemic treatment

**FIGURE 3:**
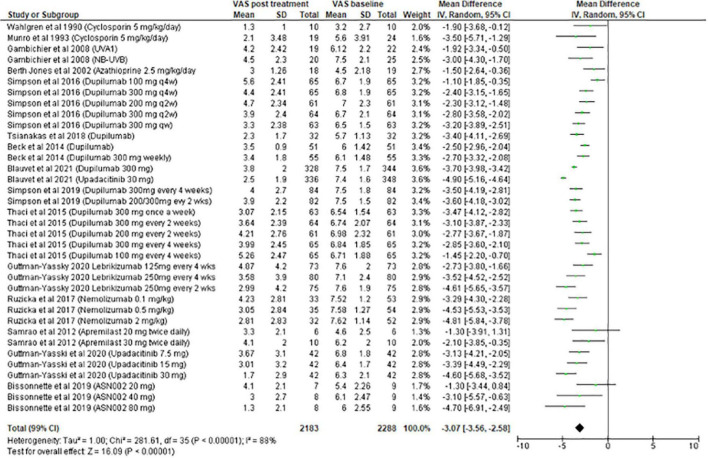
Meta-analysis on systemic treatments, without TCS (15 studies).

**FIGURE 4:**
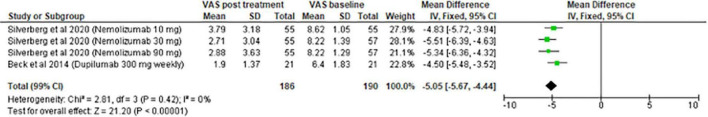
Meta-analysis on systemic treatments, with TCS (two studies).

The meta-analysis for systemic treatments included 17 articles and was divided according to the concomitant application of TCS (Figure 3) or not (Figure 4). The summary of the characteristics of the 17 studies included is presented in Table 2. In the meta-analysis without TCS (Figure 4), the global reduction in pruritus score was 3.07 points (99% CI [2.58–3.56]). In the meta-analysis of systemic treatments associated with the use of TCS (Figure 4), the mean reduction in pruritus score was 5.05 points (99% CI [4.44–5.67]). In the Supplementary Data, the meta-analysis separated by molecules are presented (Supplementary Figure 1: dupilumab, Supplementary Figure 2: lebrikizumab, Supplementary Figure 3: nemolizumab, and Supplementary Figure 4: upadacitinib).

**TABLE 2:** Summary of the characteristics of the studies included in the meta-analysis on systemic treatments.

References	Treatment	Study design	Evaluation of pruritus	Results	Time of evaluation
Wahlgren et al. (30)	5mg/kg/day of cyclosporin A (CSA) oral solution or placebo.	Double-blinded, randomized, placebo-controlled, cross over design	VAS 0–10 cm	VAS at baseline : 3.2 VAS post treatment : 1.3	10 days
Munro et al. (32)	5mg/kg/day of cyclosporin	Double-blind, randomized, placebo-controlled, cross-over study	VAS 0–10cm	At baseline, mean itch score : 5.6 At the end of treatment, mean itch score : 2.1	8 weeks
Gambichier et al. (39)	Medium-dose UVA1 versus narrowband (NB) UVB phototherapy	Monocentric, randomized, double-blinded, controlled two-treatment two-period crossover trial	VAS 0–10 cm	At the end of treatment, pruritus decreased from 6.12 to 4.2 in the group UVA1 and from 7.5 to 4.5 in the group NB-UVB.	6 weeks
Berth-Jones et al. (41)	2,5 mg/kg/day of azathioprine	Double-blinded, randomized, placebo-controlled, crossover trial	VAS 0–10cm	At week 12 (end of the first period), the mean score of pruritus in the azathioprine-placebo sequence had decreased from 4.5 to 3.0	12 weeks
Beck et al. (11)	Dupilumab evaluated as monotherapy in two 4-week trials and in one 12-week trial and in combination with topical glucocorticoids in another 4-week study	Randomized, double-blinded, placebo-controlled trials.	Pruritus NRS 0–10	4-Wk Monotherapy Change in pruritus NRS: −18.6% with placebo and −41.3% with dupilumab 12-Wk Monotherapy Change in pruritus NRS: −15.1% with placebo and −55.7% with dupilumab 4-Wk Combination Therapy Change in pruritus NRS at day 29 : −24.7% with placebo and −70.7% with dupilumab	4 and 12 weeks
Thaçi et al. (43)	Dupilumab: −300 mg once a week −300 mg every 2 weeks −200 mg every 2 weeks −300 mg every 4 weeks −100 mg every 4 weeks or placebo once a week	Phase 2, multicenter, randomized (1:1:1:1:1:1), placebo-controlled, double-blinded, parallel-group study	Peak weekly pruritus NRS 0–10	Mean peak weekly pruritus NRS score, at baseline and week 16: Dupilumab 300 mg once a week : 6.54, W16: 3.07 Dupilumab 300 mg every 2 weeks : 6.74, W16: 3.64 Dupilumab 200 mg every 2 weeks : 6.98, W16: 4.21 Dupilumab 300 mg every 4 weeks : 6.84, W16: 3.99 Dupilumab 100 mg every 4 weeks : 6.71, W16: 5.26 Placebo : 6.34, W16: 6.05	16 weeks
Simpson et al. (44)	Dupilumab −100 mg every 4 weeks −200 mg every 2 weeks −300 mg every 4 weeks −300 mg every 2 weeks −300 mg once weekly or placebo	International, randomized, placebo- controlled, double-blinded, parallel group dose-ranging study	Peak weekly pruritus NRS 0–10	Mean peak weekly pruritus NRS score, at baseline and week 16 Placebo : 6.3 Dupilumab 100 mg q4w : 6.7, W16: 5.6 Dupilumab 300 mg q4w : 6.8, W16: 4.4 Dupilumab 200 mg q2w : 7.0, W16: 4.7 Dupilumab 300 mg q2w : 6.7, W16: 3.9 Dupilumab 300 mg qw : 6.5, W16: 3.3	16 weeks
Tsianakas et al. (48)	Dupilumab 300 mg weekly or placebo	Phase II, multicenter, randomized, double-blinded, placebo-controlled trial.	Pruritus NRS 0–10	At week 12, LS mean % difference versus placebo : −50.5%, *p* < 0.001	12 weeks
Simpson et al. (49)	Dupilumab : 200 mg if <60 kg or 300 mg if ≥60 kg every 2 weeks, 300 mg every 4 weeks or placebo	Multicenter, randomized (1:1:1), double-blinded, parallel-group, phase 3 clinical trial	PP-NRS 0–10	Least-squares mean % change from baseline to week 16 in PP-NRS :**** −47.9% for dupilumab every 2 weeks −45.5% for dupilumab every 4 weeks −19.0% for placebo	16 weeks
Silverberg et al. (53)	Tralokinumab 300 mg or placebo every 2 weeks with TCS as needed over 16 weeks.	Phase 3, multicenter, double-blinded, randomized (2:1) trial	Weekly average of worst daily pruritus NRS score (0–10)	Weekly average of worst daily pruritus NRS reduction ≥ 4-point, at week 16 : Placebo + TCS : 34.1% of patients Tralokinumab + TCS : 45.4% of patients, *p* = 0.037	16 weeks
Simpson et al. (54)	Lebrikizumab 125 mg or 250mg single dose or lebrikizumab 125 mg/placebo every 4 weeks for 12 weeks	Multicenter, randomized (1:1:1:1), placebo-controlled, double-blinded, phase 2 study	VAS (0–10)	Mean VAS pruritus, % change from baseline. Lebrikizumab 125mg single dose : −34.9% (*p* = 0.40) Lebrikizumab 250mg single dose : −32.8% (*p* = 0.54) Lebrikizumab 125mg : −40,7% (*p* = 0.13) Placebo : 27.5%	12 weeks
Guttman-Yassky et al. (55)	Lebrikizumab 125 mg every 4 weeks, lebrikizumab 250 mg every 4 weeks or lebrikizumab 250 mg every 2 weeks or placebo	Phase 2b, multicenter, double-blinded, placebo-controlled, randomized clinical trial.	Pruritus NRS 0–10	Mean pruritus NRS score, at baseline and week 16: Lebrikizumab 125 mg every 4wk : 7.6 and W16: 4.87 Lebrikizumab 250 mg every 4wk : 7.1 and W16: 3.58 Lebrikizumab 250 mg every 2wk : 7.6 and W16: 2.99	16 weeks
Ruzicka et al. (56)	0.1mg/kg, 0.5mg/k or 2.0mg/kg of nemolizumab or placebo every 4 weeks	Multicenter, phase 2, randomized, double-blinded, placebo-controlled	VAS 0–10 cm	At baseline, score on pruritus visual-analogue scale and week 12: Nemolizumab 0,1mg/kg : 7.52 and W12: 4.23 Nemolizumab 0,5mg/kg : 7.58 and W12: 3.05 Nemolizumab 2mg/kg : 7.62 and W12: 2.81	12 weeks
Silverberg et al. (57)	10mg, 30mg, 30mg of nemolizumab or placebo every 4 weeks	Randomized, double-blinded, placebo-controlled, multicenter study	Peak Pruritus NRS 0–10	At baseline, peak pruritus score and week 24: 10mg of nemolizumab : 8.62 and W24: 3.79 30mg of nemolizumab :8.22 and W24: 2.71 90mg of nemolizumab 8.22 and W24: 2.88	24 weeks
Guttman-Yassky et al. (55)	7.5mg, 15mg or 30mg of upadacitinib once daily or placebo	Multicenter, phase 2b,randomized (1:1:1:1), double-blinded, placebo-controlled, parallel-group trial	Pruritus NRS 0–10	Pruritus NRS mean, at baseline and week 16 Upadacitinib 7,5 mg : 6,8 and W16: 3.67 Upadacitinib 15 mg : 6,4 and W16: 3.01 Upadacitinib 30 mg : 6,3 and W16: 1.7	16 weeks
Bissonnette et al. (25)	ASN002 20 mg or ASN002 40 mg or ASN002 80 mg once daily or placebo	Phase Ib, multicenter, randomized, double-blinded, placebo-controlled study	Pruritus NRS 0–10	Pruritus NRS at baseline and at 28 days: ASN002 20 mg : 5.4 and D28: 4.1 ASN002 40 mg : 6.1 and D28: 3 ASN002 80 mg : 6 and D28: 1.3	28 days
Samrao et al. (69)	Apremilast, 20 mg twice daily or apremilast, 30mg twice daily	Open-label pilot study	VAS 0 to 10 cm	At baseline, and 3 months: 20 mg apremilast : 4.6 cm and the end: 3.3 cm (*p* = 0.02) 30 mg apremilast: 6.2cm and the end: 4.1cm (*p* = 0.03)	3 months

## Discussion

Concerning topical treatments for AD, five studies were included in the meta-analysis. All treatments had a beneficial impact on pruritus. The most significant reduction was found for wet-wrap therapy using halometasone (−4.75 points) followed by tofacitinib 2% (−4.38 points). Although TCS are used for a long duration to treat AD and for all severity stages, studies evaluating their effect on pruritus are scarce (9). This could be explained by the fact that studies published before 1990 were not included in the present systematic review and because pruritus was less studied before.

For systemic treatments, the meta-analysis included 17 studies and was divided into 2 parts according to the use of TCS or not as concomitant treatment. In real life, TCS are associated with systemic treatments and their benefits for alleviating pruritus are obvious. Among the systemic treatments, upadacitinib 30 mg was the most effective at reducing pruritus (−4.90 points), followed by nemolizumab 2 mg/kg (−4.81 points).

Nemolizumab has a major effect on pruritus; thus, this treatment is also under development in chronic prurigo, with a phase II randomized controlled trial demonstrating efficacy compared with placebo, and 2 phase III trials are also in progress (70). However, its efficacy on AD lesions is lower than other biotherapies. In a clinical trial evaluating nemolizumab 30 mg + TCS, EASI 75 was achieved by 27% of patients at week 4, whereas it was achieved by 62% of patients at week 4 in a study with dupilumab + TCS (11, 57). In other studies, at week 16, EASI 75 was achieved by 61.1% of patients with dupilumab 300 mg and by 71% of patients with upadacitinib 30 mg, whereas in a trial that evaluated nemolizumab 2 mg/kg, only 22.3% of patients achieved EASI 75 at week 12 (56, 66). As the efficacy on AD lesions and pruritus is not always parallel, these 2 criteria are critical and can be part of the treatment decision.

To appreciate the efficacy between molecules, this study conducted a meta-analysis of the main biotherapies. For nemolizumab, combining the results revealed an improvement in the decrease of pruritus by 4.21 points. Thus, this molecule would be the most effective at reducing pruritus compared with upadacitinib (4.08 points), lebrikizumab (3.62 points), and dupilumab (2.82 points).

In addition to effectiveness on pruritus, the speed of action is interesting to analyze. Jak inhibitors are known to have a rapid effect on pruritus. For example, in a randomized trial comparing upadacitinib with placebo, Guttman-Yassky et al. highlighted that pruritus improved significantly as early as the first assessment in week 1 (64). For lebrikizumab, differences vs. placebo-treated patients of at least four-point in the pruritus NRS score were observed as early as day 2 in 3 lebrikizumab groups (55).

Anti-histamines drugs are not mentioned in this literature review because the aim was to include clinical trials evaluating treatments for AD lesions. Anti-histamines are frequently used to treat pruritus in AD whereas their efficacy on pruritus in AD was not demonstrated compared with placebo. Hydroxyzine represents a common sedating H1-antihistamine choice. However, the possible effects of these drugs should be discussed with the parents and long-term use of sedating antihistamines that is not recommended in children.

A position paper was recently published on the management of pruritus in AD, which highlighted the importance of this symptom (14). The experts recommended AD treatment, emollients and general principles, therapeutic education, and psychological support; in the case of persistent itch, they proposed discussing ciclosporin, dupilumab, nemolizumab, gabapentinoids, antidepressants, Jak inhibitors, anti-PDE4, κ opioids, or anti-NK1. In this paper, pain was also discussed. It is a different sensation from itch (characteristics, treatment) but the two sensations often coexist and it is important also to detect and treat pain.

The main limitation of this meta-analysis was the limited number of studies that it included. Among all of the clinical trials retained in the systematic review, many of them did not measure the mean change ± SD in pruritus score from baseline to the evaluation of the primary end point. Surprisingly, some recent studies did not assess pruritus (71). Moreover, different methods were used to evaluate pruritus (VAS, NRS, and semiquantitative scale), and the time and frequency of evaluating pruritus differed among the studies. Pruritus was most often evaluated as a secondary objective and not as a primary outcome, except in trials evaluating nemolizumab (56, 58).

We chose to include only patients aged 10 years or older because evaluating pruritus in children is complicated. That is, it depends on their age and few tools are available. In a phase II trial, dupilumab was studied in children aged 6 months to <6 years, and pruritus was evaluated using a caregiver-reported PP-NRS, thus creating a potential bias (72). Questionnaires must be developed for specific age groups given the differences that exist in cognitive levels. Recently, a pruritus severity instrument named ItchyQuant, which is a self-reported illustrated NRS, was developed for children aged 6–7 years with chronic pruritus (73).

In the present systematic review, two-thirds of the included studies were published in or after 2010. The majority of trials studied biotherapies and recent molecules available in AD treatment. This highlighted that available treatments for AD have multiplied in the last few years as well as that pruritus increasingly appears to be one of the main patient-reported outcomes to relieve.

The placebo effect is critical in numerous diseases and symptoms, especially in pruritus. Therefore, placebo-controlled studies are required to evaluate the placebo effect on the pruritus. In a meta-analysis evaluating the placebo effect in randomized controlled trials for different skin diseases, the placebo effect on itch in AD was evaluated as 0.75 points on a scale from 0 to 10 (95% CI [0.12–0.39]) in 10 studies (74). Although placebo-controlled trials are useful, randomized head-to-head clinical trials are also essential for comparing the efficacy of molecules on AD lesions and pruritus; an example is the recent study of Blauvelt et al., who assessed the efficacy of upadacitinib vs. dupilumab (66).

As pruritus is a major symptom impacting quality of life in AD, the efficacy of the treatment on pruritus should be considered together with the efficacy on AD lesions. The choice of treatment will also depend on the tolerance and side effects of each molecule, which have not been analyzed here.

## Conclusion

As pruritus is a subjective feeling, the objective measurement of its intensity remains challenging. Nevertheless, by focusing on the pruritus score, this meta-analysis was able to provide a quantitative estimation of the efficacy of treatments on pruritus, while the systematic review demonstrated that most topical and systemic therapies are effective at reducing pruritus. The efficacy on AD lesions and pruritus is not always parallel, thus, analyzing the pruritus score and not only the effect on AD lesions is essential. Since many therapies are now available, the effect of a treatment on pruritus could be an element in selecting a particular treatment.

## Data availability statement

The original contributions presented in this study are included in the article/Supplementary material, further inquiries can be directed to the corresponding author.

## Author contributions

YR-L, EB, and LM contributed to the conception and design of the systematic review. YR-L and EB analyzed the manuscript and wrote the first draft of the manuscript. A-SF performed the statistical analysis. All authors contributed to the manuscript revision, read, and approved the submitted version.

## Supplementary material

Supplementary Figure 1 Meta-analysis on dupilumab.

Supplementary Figure 2 Meta-analysis on lebrikizumab.

Supplementary Figure 3 Meta-analysis on nemolizumab.

Supplementary Figure 4 Meta-analysis on upadacitinib.
