# Supplementary figures and images for: Efficacy of topical and systemic treatments for atopic dermatitis on pruritus: A systematic literature review and meta-analysis
Source: Front Med (Lausanne). 2022 Dec 22;9:1079323. doi: 10.3389/fmed.2022.1079323 (PMC9814490; doi:10.3389/fmed.2022.1079323)

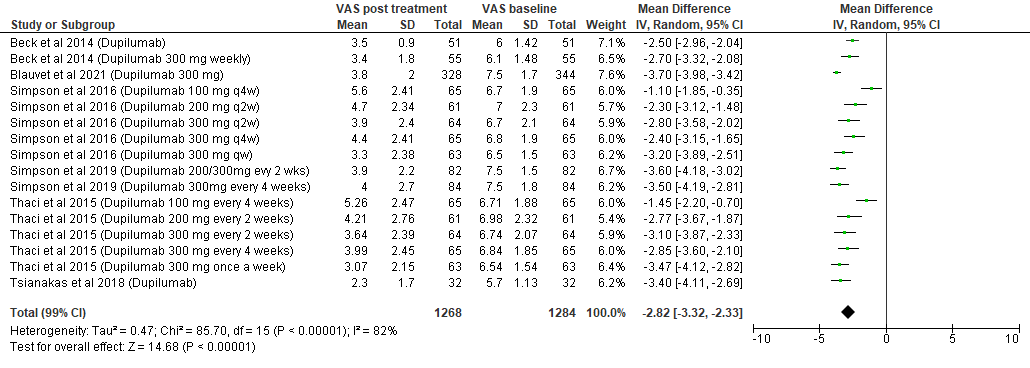

Supplement: Supplementary Figure 1 — Meta-analysis on dupilumab. [file Image_1.tif]

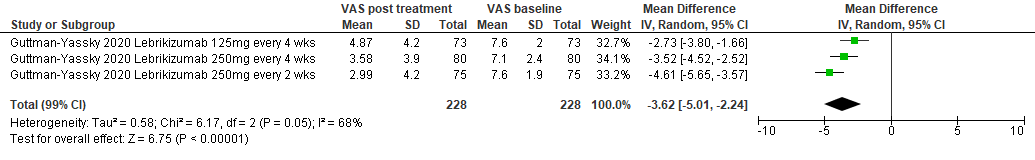

Supplement: Supplementary Figure 2 — Meta-analysis on lebrikizumab. [file Image_2.tif]

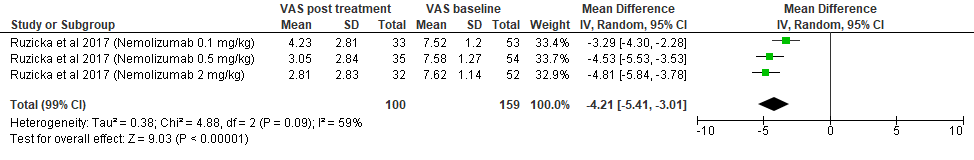

Supplement: Supplementary Figure 3 — Meta-analysis on nemolizumab. [file Image_3.tif]

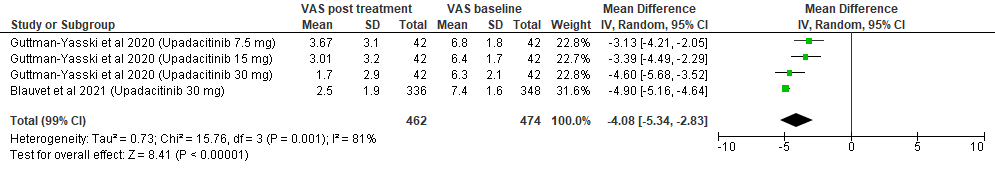

Supplement: Supplementary Figure 4 — Meta-analysis on upadacitinib. [file Image_4.tif]
